# Sexual dimorphism of colorectal cancer in humans and colorectal tumors in a murine model

**DOI:** 10.3389/fonc.2024.1398175

**Published:** 2024-08-06

**Authors:** Yair Rodríguez-Santiago, Luis Ignacio Terrazas-Valdés, Karen Elizabeth Nava-Castro, Víctor Hugo Del Río-Araiza, Claudia Angélica Garay-Canales, Jorge Morales-Montor

**Affiliations:** ^1^ Departamento de Inmunología, Instituto de Investigaciones Biomédicas, Universidad Nacional Autónoma de México, Ciudad Universitaria, Ciudad de México, Mexico; ^2^ Posgrado en Ciencias Biológicas, Universidad Nacional Autónoma de México, Ciudad Universitaria, Ciudad de México, Mexico; ^3^ Unidad de Biomedicina, Facultad de Estudios Superiores Iztacala, Universidad Nacional Autónoma de México, Iztacala, Tlanepantla, Mexico; ^4^ Laboratorio Nacional en Salud, Facultad de Estudios Superiores Iztacala, Universidad Nacional Autónoma de México, Iztacala, Tlanepantla, Mexico; ^5^ Laboratorio de Genotoxicología y Medicina Ambientales, Departamento de Ciencias Ambientales, Instituto de Ciencias de la Atmósfera y Cambio Climático, Universidad Nacional Autónoma de México, Ciudad de México, Mexico; ^6^ Laboratorio de Interacciones Endocrinoinmunitarias en Enfermedades Parasitarias, Facultad de Medicina Veterinaria y Zootecnia, Departamento de Parasitología, Universidad Nacional Autónoma de México, Ciudad de México, Mexico

**Keywords:** colorectal cancer, sexual dimorphism, sex steroids, estradiol, dihydrotestosterone

## Abstract

**Introduction:**

In colorectal cancer, men exhibit a higher incidence than women, and there is a disturbance in the levels of sex steroids in serum in patients with this disease. Consistently, in animals, males have greater tumor growth than females in diverse models. Nevertheless, the role of sex steroids is not well established. For that, we analyzed the effect of the principal gonadal sex steroids in both sexes. We determined sex as a statistically risk factor for colorectal cancer with data obtained from GLOBOCAN database.

**Methods:**

To induce colorectal tumors, we used the gold standard chemical method of azoxymethane and dextran sulphate of sodium. To evaluate the role of sex steroids, we gonadectomized independent males and female animals, reconstituting and substituting them with 17β estradiol and dihydrotestosterone. Finally, we determined, in vitro, the proliferation of a human cell line exposed to 17β estradiol, testosterone, or dihydrotestosterone. Sex, as a risk factor for colorectal cancer, showed a statistically significant susceptibility of men over 50 years old.

**Results:**

In vivo, males develop a greater number of tumors and with a larger size than females. In males, orchiectomy prevents tumor growth, whereas in females, ovariectomy promotes the development of neoplasms. DHT acts as a protumoral agent in both sexes. 17β estradiol reduces tumor growth in females but enhances it in males, showing a dimorphic effect. In vitro studies reveal that estradiol decreases the proliferation of the HCT-116 colon cancer cell line, while testosterone boosts proliferation in these cells. Interestingly, dihydrotestosterone does not influence proliferation.

## 1 Introduction

Sexual dimorphism refers to the differences in morphology and physiology between males and females of the same species. This concept is particularly important in the study of diseases, as it results in different susceptibilities for each sex, influencing mortality, morbidity, prevalence, intensity, severity, behavioral changes, and immune response (1). For instance, colorectal cancer (CRC) is the third most common heterogeneous neoplasm worldwide, with several studies reporting a higher incidence in men than in women (2). The causes of sexual dimorphism can be attributed to various factors, including gender and sex distinctions such as behavioral and physiological differences (3). Moreover, sexual dimorphism appears to persist regardless of ethnicity or geographic location, suggesting that intrinsic factors play a fundamental role in explaining these disparities (2).

The concentration of sex steroids is one of the most important intrinsic factors that differ between both sexes (4). These molecules play crucial roles in the gut: Estrogens influence epithelial membrane permeability, serotonin production, the expression of tight junctions, inflammation, and microbiome composition (5). On the other hand, the effects of androgens are not fully understood. However, in a mouse model, they have been shown to alter the proliferation of enterocytes and increase the size of crypts (6).

In colorectal cancer patients the levels of sex steroids are altered. For instance, postmenopausal women with CRC have higher levels of 17β estradiol (E2) and estrone and a greater ratio of testosterone (T4) to E2 than control patients do; however, this pattern is not observed in men (7). On another hand, the expression of aromatase, an enzyme responsible for converting T4 to E2, is greater in neoplastic tissue in both men and women. This expression is inversely associated with the proliferation index, tubular differentiation, and estrogen concentration just in men and not in postmenopausal women (8). Consistently, serum levels of E2 are greater in male patients with CRC than in healthy controls (9).

The relationship between androgens and CRC has been poorly studied. Contrary to expectations, the circulating level of T4 is inversely associated with overall survival and mortality in CRC patients but only in men (10, 11). A reduction in androgen levels is associated with fewer CAG repeats in the androgen receptor (AR) gene, which subsequently increases transcriptional activity (12). AR is overexpressed in colon tumor tissue and is associated with tumor size, differentiation, and distant metastasis (13). In contrast, this receptor is not expressed in the non-neoplastic mucosa. Interestingly, in postmenopausal women, a few CAG repeats in the AR gene and a CA repeat in the ER-β gene are associated with high serum androgen levels, suggesting that these polymorphisms have a stimulatory effect on T4 production in women (14). Although further research is needed to understand deregulation of sex steroid concentrations and its importance in the physophatology of CRC.

*In vitro* studies with colon cancer cell lines such as DLD-1, HCT-116, SW480, and CaCO2 have revealed that E2 reduces the viability of these cells, while T4 and dihydrotestosterone (DHT) increase apoptosis in CaCO2 and HCT116 (5, 15–24). E2 reduces the viability of colon cancer cells by activating P53, which subsequently upregulate the levels of p21 and p27, that consequently inhibits cyclin D1 gene that reduce proliferation (25). Additionally, the treatment with E2 decreases cellular migration by reducing MMP-2 and MMP-9 levels and inhibiting the JNK 1/2/PGE2 pathway, which is involved in cellular motility (26).

Experiments with microarrays have shown that ER-β plays an important role in the regulation of transcription factors such as MYC, MYB, RUNX2, and PROX1, which are involved in cell viability, proliferation, apoptosis, and differentiation. This modulation potentially triggers an antitumoral cascade (27). On another hand, T4 increases apoptosis in cells by reducing PI3K/Rac1, which activates the kinase JKN, stimulating the transcription of proapoptotic genes involved in intrinsic and extrinsic apoptosis (23, 28). Finally, reorganization of the cytoskeleton reduces the migration and invasion of colon cancer cells. This effect is not influenced by the inhibition of aromatase, suggesting that the effect is directly mediated by T4 and not by the conversion of T4 to E2 (29); since, this enzyme metabolizes T to E2 and E2 is a final metabolite in the steroidogenesis.

There are few *in vivo* studies in which sexual dimorphism is evident. Lee et al. described a rapid and effective method for modeling human colitis-associated cancer using the carcinogen azoxymethane (AOM) and subsequently treating ICR mice with the pro-inflammatory agent dextran sulfate sodium (DSS) (30). In this study, they observed that male mice developed more tumors than females; however, the role of sex steroids remains unclear. For instance, male PIRC rats developed twice as many adenomas as females (31); In this research, neither ovariectomy nor reconstitution with E2 in PIRC rats affected the development of adenomas. In contrast, Song et al. showed the protective effect of endogenous or E2 replacement in ovariectomized female ICR mice against AOM/DSS-induced colitis (32). In another research, males treated with estradiol develop fewer tumors and less tissue damage (33). In an inflammation context, there are few parameters that has been studied to explain the protector effect of E2 in both sexes. This hormone reduce inflammation diminishing parameters such as COX-2, IL-6, TNF-α, iNOS and IL-1β (32, 33).

In the AOM-DSS model in C57BL6 mice, T4 significantly enhances the induction of colorectal cancer (34); Conversely, orchiectomy in PIRC rats drastically reduces the number of tumors, and this effect is countered by reconstitution with DHT (31). In contrast, treatment with a conjugate of T4 and albumin significantly reduces tumor incidence induced by dimethylhydrazine and DSS in Balb/c mice (35). These apparently contradictory results could be explained by T4 potential metabolism into E2 and DHT, making it challenging to elucidate its specific effects. Furthermore, its conjugation with albumin primarily interacts with the androgen membrane receptor. The above findings underscore the importance of conducting a study with standardized conditions, to assess the involvement of sex steroids in the induction of colorectal tumors in both males and females. Therefore, the goal of this research is to determine whether sex-related susceptibility in the induction of colorectal tumors is mediated by the concentrations of the principal sex steroids (E2 and DHT), evaluated in a murine model.

## 2 Materials and methods

### 2.1 Evaluation of sex as a risk factor in Mexico and worldwide

The most recent data (2020) was obtained from the GLOBOCAN database, which it is an openly available database that contains latest data on cancer around the world (https://gco.iarc.fr/) (36). The parameters chosen were age-standardized rates of men and women over 50 and under 50 years old to evaluate the effect of age and sex in the incidence of CRC. With this data a statistical analysis was carried out (incidence relative risk, t-student analysis and confidence intervals).

### 2.2 Animals and ethics statement

Animal care and experimental practices were conducted at the Unidad de Modelos Biológicos (UMB) in the Instituto de Investigaciones Biomédicas (IIB), Universidad Nacional Autónoma de México (UNAM). All experimental procedures in the animals were approved by the Institutional Care and Animal Use Committee (CICUAL), ID Number 6298, adhering to Mexican regulation (NOM-062-ZOO-1999), and in accordance with the recommendation from the National Institute of Health (NIH) of the United States of America (Guide for the Care and Use of Laboratory Animals). Mice of the syngeneic strain Balb/c AnN were purchased from Harlan, Facultad de Química, UNAM, México. The animals were housed at the UMB under controlled temperature (22°C) and 12 hours of light-dark cycles.

### 2.3 Gonadectomy in male and female mice

Gonadectomies were performed at four weeks of age on Balb/c mice according to the protocol previously reported by Villavicencio, et al (37). Briefly, animals were anesthetized with a cocktail of xylazine and ketamine (Pisa, Atitalaquia Hidalgo, México). Ovariectomy (OVX) was carried out by making an incision in the dorsal part; the ovary was located under the muscle layer and a cut was made to enter the peritoneal cavity. The oviducts were ligated, and the ovary was removed. After removal, the incision was closed using an absorbable synthetic suture (triple zero polyglycolic acid) (Atramat, México).

For orchiectomy (ORX), an incision was performed on the ventral part of the scrotum. The testicular fat was located and gently pulled away, exposing the epididymis, vas deferens, and testis. The blood vessels were ligated, and the testicle was removed. Once both testicles were removed, the surgery was concluded by suturing the skin using an absorbable synthetic suture.

### 2.4 Reconstitution and substitution of sex steroids

To evaluate if the observed effect is through sex hormones, we assessed the effect of reconstitution and substitution of E2 and DHT. We administered sex steroids via intraepithelial injection every four days on the back of the neck of mice one week after the gonadectomy was performed. The doses were calculated with based on the work of Morales J and coworkers that use a released pellet of 0.05 mg (38). Assuming that hormone release is constant, the approximate dose of hormone that the mouse receives is ∼1.66 micrograms/day. This dose was calculated by dividing the dose used in Morales-Montor’s work by the number of days the pellet release lasts and multiplying it by four, which is the period between injections.

### 2.5 Colon tumor induction

The induction of colorectal tumors was performed following the protocol previously reported by Leon-Cabrera (39). Briefly, four weeks after gonadectomy, male and female mice: control, sham, and gonadectomized were injected intraperitoneally with 12.5 mg/kg of azoxymethane (AOM) (Sigma-Aldrich, St. Louis, Missouri, USA). Five days later, mice were treated with the pro-inflammatory agent dextran sulfate sodium DSS (Alfa Aesar, Ward Hill, Massachusetts, USA) in water ad libitum for seven days. Subsequently, there were two weeks of rest (with potable water), and the cycle was repeated three times.

### 2.6 Sacrifice and tumor processing

At day 70 from the start of AOM-DSS treatment, mice were euthanized by inhalation of a mixture of air and sevoflurane (5%) followed by cervical dislocation. Blood was drawn by cardiac puncture to obtain the serum. Subsequently, the colon of each mouse was removed and cut to expose the tumors which were then placed on graph paper. First, we counted the total number of tumors, and then we evaluated their size by identifying tumors larger than two millimeters. The analysis was carried out on millimeter paper to have a size reference. Additionally, we analyzed the tumor burden score (TBS), taking into account the number and size of tumors with the following formula: TBS^2^ = (maximum tumor diameter)^2^ + (number of tumors)^2^, which is associated with the prognosis of the disease.

### 2.7 Quantification of concentration of sex steroids

The sex steroids were extracted using an ether-methanol method, and their concentrations were calculated using ELISA kit for E2, T4 (Arbor Assays, Ann Arbor, Michigan, USA), and DHT (Eagle Bioscience, Nashua, New Hampshire, USA), following the manufacturer’s instructions.

### 2.8 Analysis of proliferation in colon cancer cell line HCT116

The HCT-116 cell line was obtained from the American Type Culture Collection (ATCC), the world’s premier biological culture repository. Cells were cultured in RPMI 1640 medium (Sigma, St. Louis, Missouri, USA) supplemented with 10% of FBS, glutamine, antibiotics, sodium pyruvate, and essential amino acids (GIBCO, Invitrogen, Grand Island, New York, United States). Cells were counted in a Neubauer Chamber and placed in 96-well culture plate at 1500 cells/well cultured with RPMI free of sex steroids and phenol red for 24 hours. Subsequently, increasing concentrations of E2, T4, and DHT from 10^-10^ to 10^-5^ M were added to corresponding wells. After an additional 72 hours, proliferation was determined with CyQUANT KIT following the manufacturer’s protocol in a Cytation 1 Cell Imaging Multi-Mode Reader (Biotek, Santa Clara CA, United States). The appropriate number and time to evaluate the effects of sex steroids were determined by a proliferation curve o using the sulphorodamine method (Data not shown).

### 2.9 Analysis of proliferation by blocking sex steroid receptors

We assessed the effect of estrogen receptor agonists ER-α (PPT) and ER-β (ER-β 041), as well as antagonists of ER-α (MPP) and ER-β (PHTPP) at an equimolar concentration of E2 (1x10^-6^), purchased from TOCRIS Bioscience, on proliferation after 72 hours of treatment. Additionally, we analyzed the effect of flutamide, an androgen receptor agonist purchased from Sigma Aldrich, at an equimolar concentration of T4 (1x10^-10^). All the experiments were evaluated after an additional 72 hours, and proliferation was determined using the CyQUANT KIT following the manufacturer’s protocol and a Cytation 1 Cell Imaging Multi-Mode Reader (Biotek, Santa Clara CA, United States).

### 2.10 Statistical analysis

To evaluate sex as a risk factor, the relative risk was calculated based on the data obtained from the GLOBOCAN database (2020). The parameters we chose were: age, patients under and older 50 years old; sex, men and women; cancer type, colorectum; population parameter, Age standardized risk of each region. Then, student T, relative risk and confidence intervals were calculated to assess significance. First, we use a T of student, since we compared two groups of data. Subsequently, we calculated the relative risk considering the sex such as an exposition factor, and, when relative risk is calculated to estimate if this relative risk is significative, confidence intervals must be calculated.

Data from 2-3 independent experiments are presented as mean +/- standard deviation and analyzed with Prism 5 software (GraphPad Software Inc.). Subsequently, one-way ANOVA was performed, followed by a Tukey *post-hoc* test. The difference was considered significant when P< 0.05. We chose ANOVA since we have multiple independent groups and subsequently a pos hoc tukey since we needed to compare samples in pairs, for example, sham males vs Gx males.

## 3 Results

### 3.1 Evaluation of sex as a risk factor in humans

**Figure 1:**
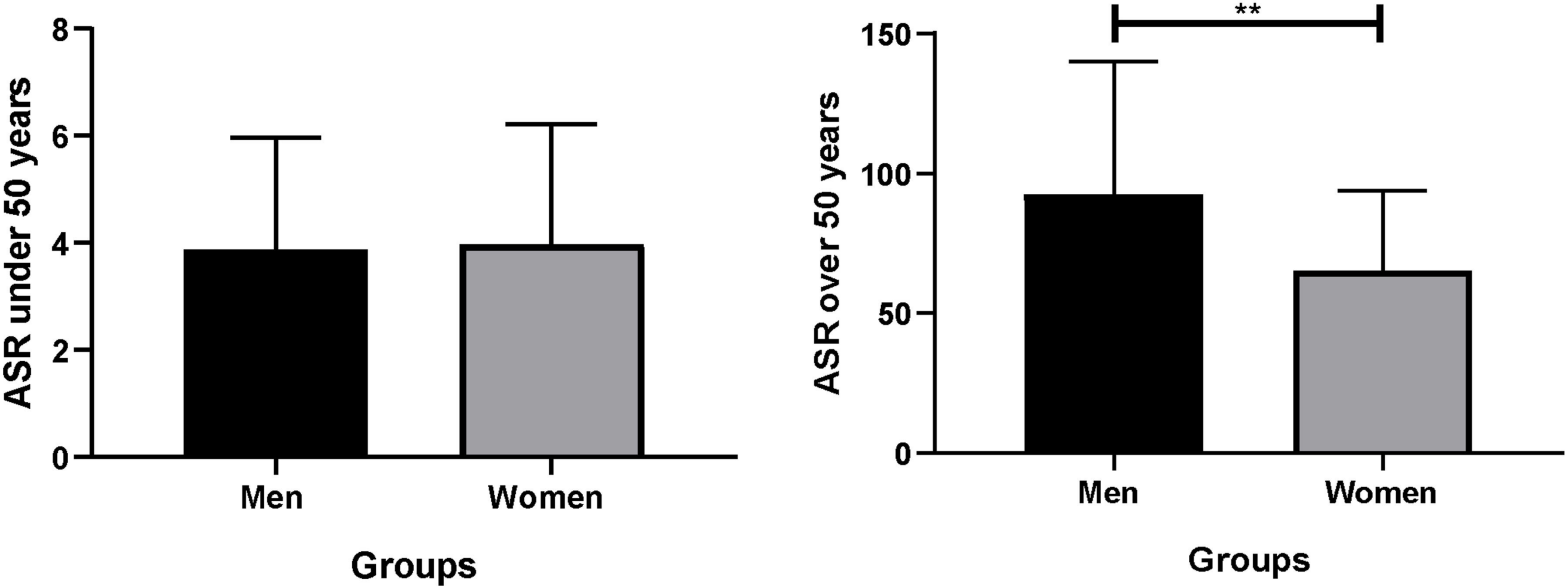
Analysis of the incidence of CRC by age. ASR was standardized for 100,000 individuals. Significance was defined with p < 0.05. ASR, age standardized rate. **(p< 0.05).

The incidence of colorectal cancer was obtained from the most recent data available from GLOBOCAN (2020) (40). The age standardized rates are calculated per 100,000 individuals. There were no significant differences between men and women under 50 years old (CI: -0.29 to 0.15). However, men over 50 years old (CI: 21.29 to 40.37) had a greater relative risk than women (1.24 times greater) of developing CRC (p = 0.040, DF = 70). This analysis confirms, through a statistical analysis, that sex is a risk factor for developing CRC, considering age (**Figure 1**).

### 3.2 Analysis of the number of tumors in males

**Figure 2:**
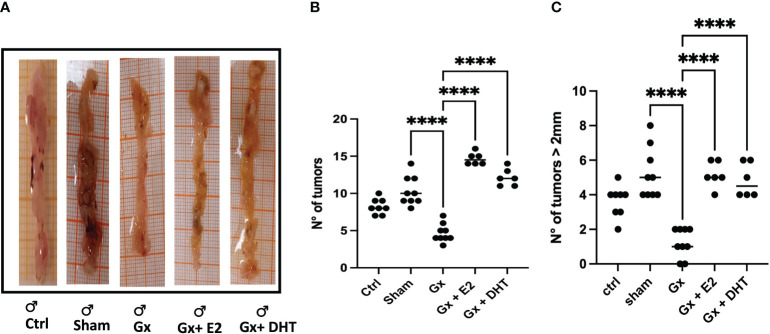
Representative photos **(A)** and statistical analysis of the number **(B)** and size **(C)** of tumors in males. Significance was defined with p < 0.05. Ctrl, control; Gx, gonadectomized; Sham, simulated surgery; E2, 17β estradiol; T4, Testosterone; DHT, dihydrotestosterone. **** (p< 0.0001).

We divided the results by sex to demonstrate the findings more clearly. Consistent with epidemiological analysis, males exhibited an increase in tumor growth compared to females (q = 7.293, DF = 27, p = 0.0001). Androgens has a protumoral role. Since, gonadectomy in males led to reduction in the number of tumors (q = 13.28, DF = 33, p < 0.0001), and the reconstitution with DHT, resulting in an increase in the number of tumors (q = 16.15, DF = 33, p < 0.0001). Unexpectedly, substitution with E2 increased neoplastic lesion (q = 21.23, DF = 33, p < 0.0001) (**Figures 2A**, **B**).

### 3.3 Analysis of size of tumors in males

In the development of colorectal cancer, tumor size is a crucial parameter. It is closely associated with the prognosis of the disease, particularly in terms of tumor grade and stage. Our results reveal that males exhibited a greater incidence of tumors larger than 2 mm compared to control females (q = 11.16, DF = 61, p < 0.0001). Orchidectomy (ORX) in males reduced drastically tumor growth (q = 11.92, DF = 33, p < 0.0001. Reconstitution with DHT increased this parameter (q = 8.714, DF = 33, p < 0.0001). As in the number, the size of tumors increased in males treated with E2 (q = 9.225, DF = 33, p < 0.0001) (**Figures 2A**, **C**).

### 3.4 Analysis of the tumor burden score in males

**Figure 3:**
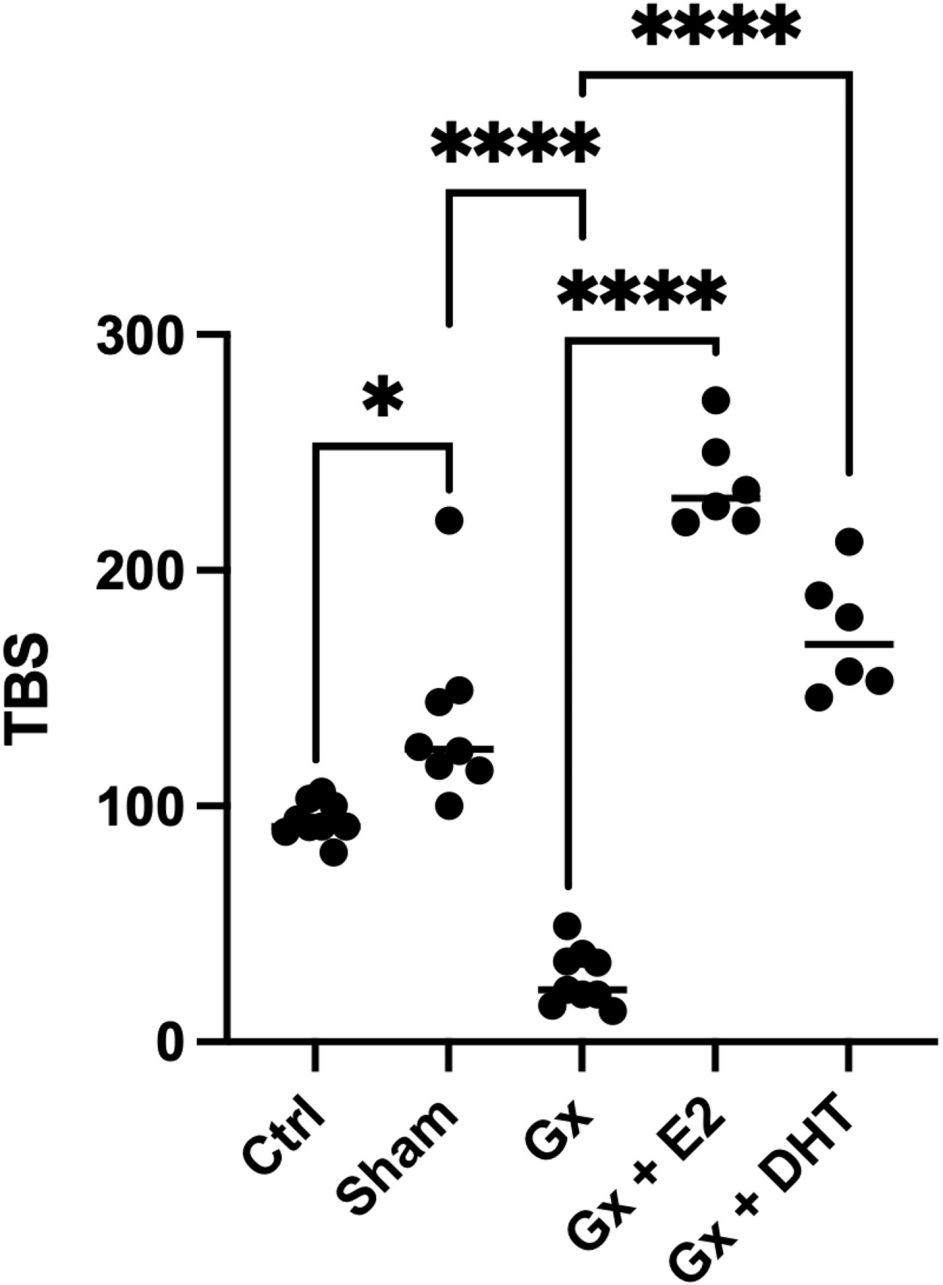
Statistical analysis of the tumor burden score in males. Significance was defined as p< 0.05. Ctrl, control; Gx,gonadectomized; Sham, simulated surgery E2,17β estradiol; T4,Testosterone; DHT, dihydrotestosterone. Tumor burden is the relation of the maximun size of tumor and the number of tumors. * (p< 0.05) and **** (p< 0.0001).

We also considered, the relationship between the numbers and the size of tumors calculated by the TBS. This parameter considers the number and the maximum tumor size. Recently, this parameter has been used as a marker in the prognosis of metastatic CRC and cholangiocarcinoma. Even, the measurement agrees with carbohydrate antigen, which is used as a prognostic and diagnostic marker for pancreatic, colorectal and gastric cancer. Interestingly, the stress of surgery plays a role in the TBS. However, the TBS is more pronounced in gonadectomized animals (q = 12.94, DF = 33, p < 0.0001). Reconstitution with DHT increases six times the TBS (q = 15.33, DF = 33, p < 0.0001), while the substitution with E2 increases eight times the TBS value (q = 21.11, DF = 33, p < 0.0001). Consistent with our analysis of numbers and sizes, the TBS suggests that E2 and DHT have a protumoral role in males (**Figure 3**).

### 3.5 Concentration of sex steroids in serum of males

Interestingly, the control and sham groups (with tumors) exhibited lower levels of testosterone than intact mice (without tumors) (q = 7.512, DF = 27, p = 0.0002) (718 vs. 117 pg/ml). However, Gx mice showed no detectable levels of T4, suggesting that gonadectomy was performed correctly. In contrast, sham and control males had higher levels of DHT (527, 466, and 345 pg/ml), while Gx mice exhibited undetectable levels of this hormone (q = 5.816, DF = 27, p = 0.0057).

**Figure 4:**
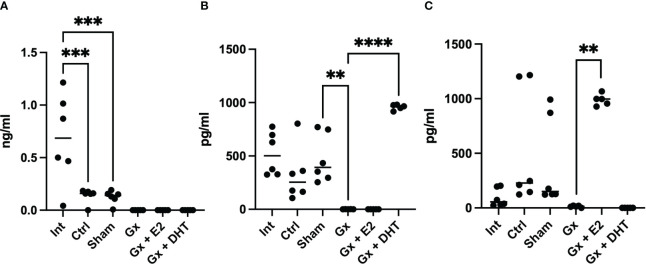
T4, DHT and E2 serum concentration of males. Significance was defined as p <0.05. Ctrl, control; Gx, gonadectomized; Sham, simulated surgery; E2,17β estradiol; T4, Testosterone; DHT, dihydrotestosterone. ** (p< 0.01), ***(p< 0.001) and ****(p< 0.0001).

The levels of E2 increased in the control and sham mice compared to the intact group without significance (q = 0.4038, DF = 27, p = 0.8214) (121.65 vs. 423.75 and 238 pg/ml). Gonadectomy tend to reduce the levels of E2 to 21.23 pg/ml but without significance (q = 3.530, DF = 27, p = 0.5151), implying that, T4 was metabolized to E2 in males with tumors (**Figure 4**).

### 3.6 Analysis of the number of tumors in females

**Figure 5:**
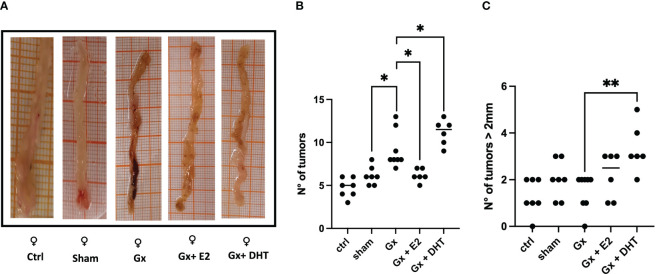
Representative photos **(A)** and statistical analysis of the number **(B)** and size **(C)** of tumors in females. Significance was defined as p < 0.05. Ctrl, control; Gx, gonadectomized; Sham, simulated surgery. * (p< 0.05) and **(p< 0.01).

Females develop fewer tumors than males (q = 7.293, DF = 27, p = 0.0001). As expected, estrogens had a protector role. ovariectomy increase the number of tumors group (q = 4.554, DF = 29, p = 0.0247), while the reconstitution with E2 reduce the growth of neoplastic lesions (q = 4.492, DF = 29, p = 0.0264). Similar to males, reconstitution with DHT has a protumoral effect (q = 5.019, DF = 29, p = 0. 01015) (**Figures 5A**, **B**).

### 3.7 Analysis of the size of tumors in females

The size of tumors was greater only in females reconstituted with DHT (q = 5.563, DF = 29, p = 0.0040) (**Figures 5A**, **C**).

#### 3.7.1 Analysis of the tumor burden score in females

**Figure 6:**
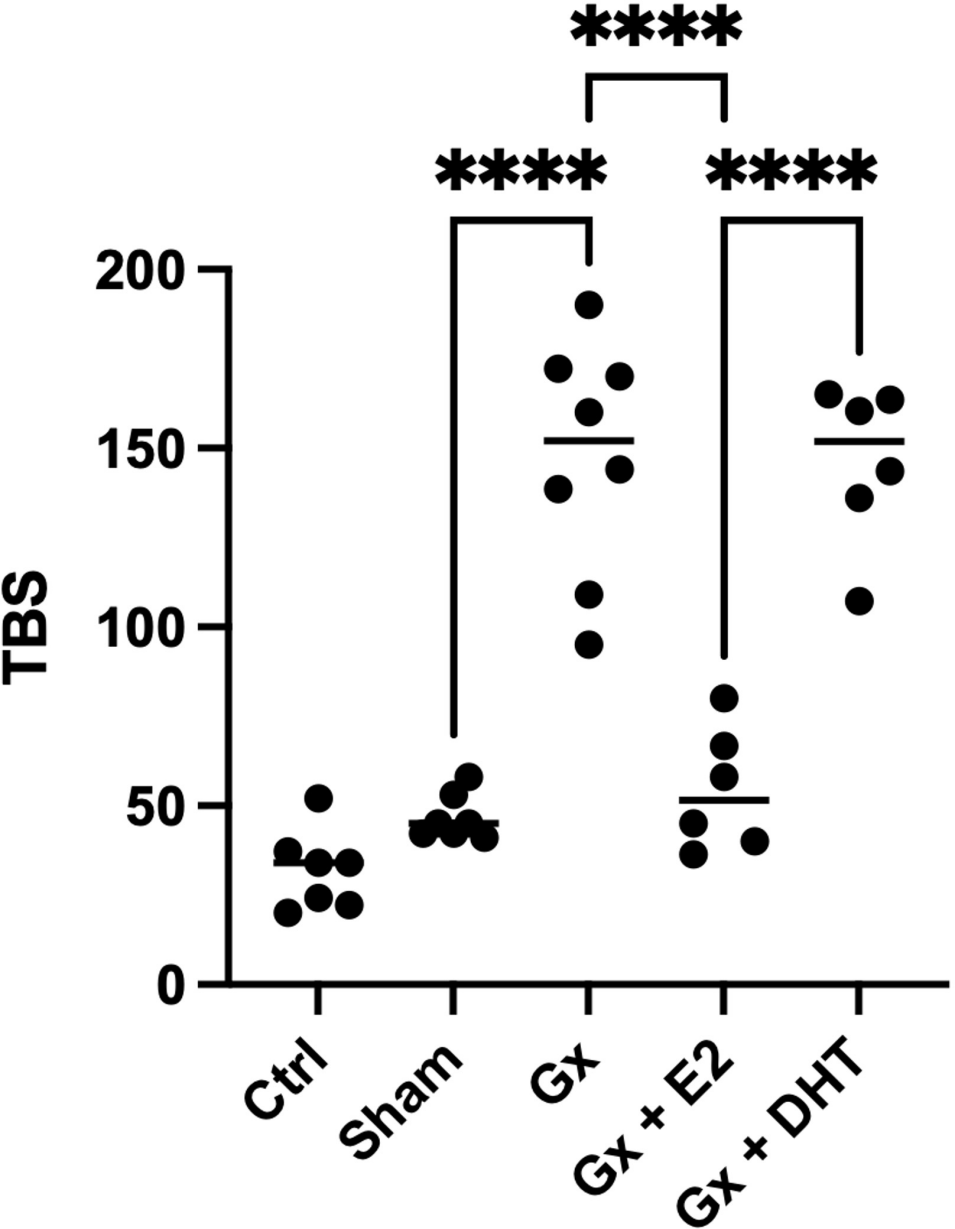
Statistical analysis of the tumor burden score in males. Significance was defined as p< 0.05. Ctrl, control; Gx,gonadectomized; Sham, simulated surgery E2,17β estradiol; T4,Testosterone; DHT, dihydrotestosterone. Tumor burden is the relation of the maximun size of tumor and the number of tumors. **** (p< 0.0001).

When we consider both the number and the maximum size of tumors. Estrogens continue to show a protective role. Since, TBS in gonadectomy group is greater than in control q = 12.94, DF = 29, p < 0.0001) and reconstituted females with E2 (q = 21. 11, DF = 29, p < 0.0001). Although substitution with DHT had no effect on TBS, it did significantly increase the number of tumors compared to the sham and Gx group, suggesting a pro-tumoral effect of this hormone in both sexes (**Figure 6**).

### 3.8 Sex steroids concentration in serum in females

**Figure 7:**
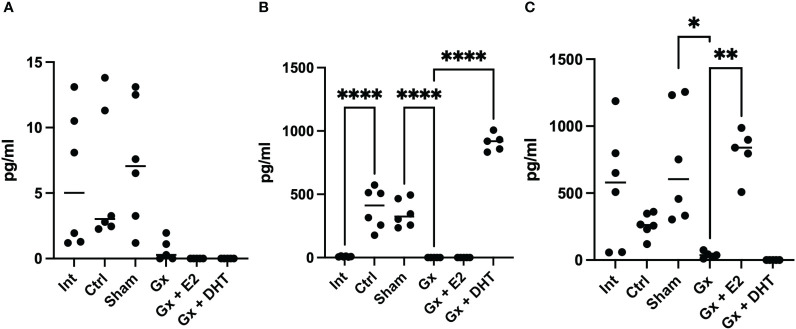
T4, DHT and E2 serum concentration of females. Significance was defined as p <0.05. Ctrl, control; Gx, gonadectomized; Sham, simulated surgery; E2,17β estradiol; T4, Testosterone; DHT, dihydrotestosterone. * (p< 0.05) **(p*< 0.01) and ***(p< 0.001).

In females, the intact, control, sham, and Gx groups did not show any changes in the levels of testosterone (6.093 pg/ml). Interestingly, the control and sham groups (with tumors) had higher levels of DHT compared to intact females (355 vs. 7.59 pg/ml) (q = 9. 953, DF = 27, p < 0.0001), while Gx females had no detectable values of this hormone. This suggests that T4 is being biotransformed into DHT (**Figure 7**).

### 3.9 *In vitro* proliferation of colon cancer cell lines

**Figure 8:**
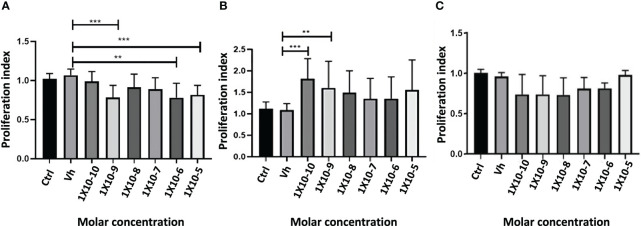
Proliferation index of the exposition of HCT116 cell with 17β estradiol E2 **(A)**, Testosterone T4 **(B)** and Dihydrotestosterone DHT **(C)**. Significance was defined as p < 0.05. Ctrl, control; Veh, vehicle. **(p< 0.01) and ***(p< 0.001).

The proliferation was determined with CyQuant kit, after 72 hours of exposure to HCT-116 cells with sex steroids (E2, T4, or DHT) or vehicle (ethanol). The treatment with E2 significantly reduces the proliferation in a dose-dependent manner. T4 increased this parameter, while, unexpectedly, DHT not influenced cell proliferation (**Figure 8**).

### 3.10 Effect of blocking sex-steroid receptors on HCT116 cell line

**Figure 9:**
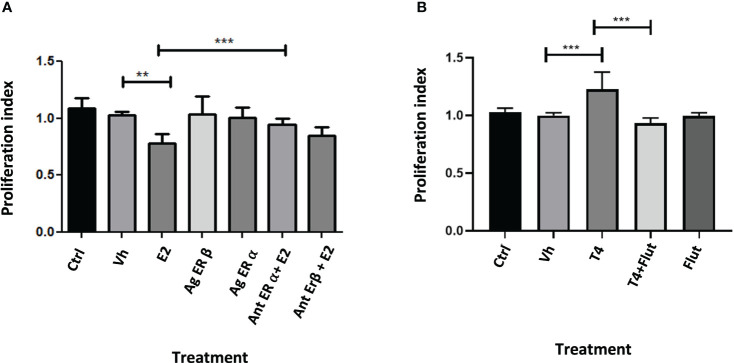
Proliferation index of the exposure of HCT116 cell with agonists and antagonists of estrogen receptors **(A)** and with an inhibitor of androgen receptor **(B)**. Significance was defined as p < 0.05. Ctrl, control; Veh, vehicle; Ag ER Agonist of ER-α or ER-β; Antagonist of ER-α or ER-β. Agonist and antagonist were administrated at equimolar concentration with sex steroids evaluated. Effect of blocking sex-steroid receptors on HCT116 cell line< 0.05. Ctrl, control; Gx, gonadectomized; Sham, simulated surgery. **(p< 0.01) and ***(p< 0.001).

To evaluate if the effect of E2 and T4 is mediated through sex steroid receptors, we treated HCT116 with agonists and antagonists of estrogen receptors and with one antagonist of androgen receptor (the cotreatment was realized in a equimolar concentration). The antagonist of ER-β (HTPP) inhibits the effect of E2, while the antagonist of the androgen (flutamide) receptor inhibits the effect of T4. These findings suggest that E2 protects against CRC through ER-β, while the proliferative effect of androgens is mediated through cytoplasmic androgen receptor (**Figure 9**).

## 4 Discussion

In humans, CRC is one of the most common malignant neoplasms, ranking 2nd to 4th in terms of incidence depending on factors such as location, cancer type, or gender. The study of risk factors is highly relevant due to the increasing incidence in terms of morbidity and mortality. Several factors may be responsible for the development of this disease. The progression from adenoma to carcinoma exhibits clear sexual dimorphism, with a preference for male development of both adenomas and CRC (31). Therefore, we undertook a study to investigate the role of sex hormones in CRC progression. We used a murine model widely accepted through the AOM-DSS method. Gonadectomy was performed to evaluate the influence of sex steroids in this process, reconstituting and substituting with the principal sex steroids (E2 and DHT).

As expected, the induction of tumors showed a dimorphic pattern. Males exhibited greater growth of tumors in size and number, and a significantly higher TBS compared to females. This parameter considers the number and the maximum tumor size in the tissue. In addition, recent studies have shown that tumor burden score is a good marker for prognosis and is associated with some molecular mutations such as KRAS (41).

Consistent with our findings, previous studies involving ICR, C57BL/6 and APC/min male mice had shown a higher incidence of tumors compared to females (31, 33, 34). In our results, gonadectomy in females increased the number of neoplasias and resulted in a higher TBS than the sham group. In contrast, in males, orchiectomy reduced the number of tumors, lowered the fold change in tumors larger than 2 mm and decreased the TBS value. This demonstrates a greater effect on the fold change in the number, size, and TBS of tumors in males undergoing orchiectomy compared to females undergoing ovariectomy. This observation is particularly relevant, as not only the number but also the size of tumors and their correlation (TBS) in CRC are closely associated with prognosis, including tumor stage and metastasis.

This suggests that androgens play a more significant role in the progression of colorectal tumors compared to estrogens, at least in males (42, 43). Although DHT levels are not modified in intact males compared to control male mice, the levels of T4 were lower in control animals (with tumors). This suggests that DHT is responsible for the protumoral effect. In line with this, the administration of DHT in both sexes favored the growth of tumors compared to gonadectomized groups. Consistently with our results, orchiectomy in PIRC rats, which are spontaneously susceptible to developing adenomas, reduced the formation of neoplastic lesions, and reconstitution with DHT increased the number of tumors (31). These observations open the possibility of using drugs that inhibit the interaction of androgens with their receptor, such as flutamide or apalutamide, both of which are approved for the treatment of prostate cancer.

The decrease in T4 levels and the increase in the concentration of E2 in control males, when compared with intact mice, suggest that T4 is being converted to E2. Consequently, the serum concentration of T4 in patients with CRC is lower compared to control cases. Additionally, CRC patients exhibit a higher expression of aromatase (enzyme that convert T4 in E2) in neoplastic tissue associated with E2 in men (8, 44). Interestingly, the lower concentration of circulating T4 is associated with overall survival, although the expression of androgen receptors is directly associated with tumor size, tumor differentiation, and distant metastasis (11, 45). Unexpectedly, the concentration of DHT was higher in control females (with tumors) compared to the intact group. However, ovariectomy significantly decreases the levels of this hormone to undetectable levels. On the other hand, T4 levels are reduced in ovariectomized females, suggesting that T4 is being converted to DHT. Although the substitution with DHT increases the number of tumors by 1.4 times compared to Gx females, the concentration of DHT is higher compared to control animals. This suggests that the biological effect on tumor growth depends on the dose. Another possible explanation is that E2 is the principal factor that plays a role in the development of colorectal tumors in females.

Ovariectomized females had lower levels of E2 compared to intact and control mice. Ovariectomy increased the number of tumors and the TBS relation. Reconstituted females exhibited a decrease in the number of tumors and the TBS value. These findings suggest that estrogens play an antitumoral role, in alignment with epidemiological, *in vitro*, and *in vivo* studies. For instance, hormonal therapy replacement and the consumption of foods rich in phytoestrogens reduce the risk of developing CRC in postmenopausal women. Unfortunately, HTR is associated with a higher risk of breast cancer (46–48). Estrogens favor the proliferation and mutation of breast cancer cells by the interaction with the estrogen receptor alpha (48). In contrast, the expression of estrogen receptor β is directly associated with high survival and inversely with the stage of tumor in CRC patients (49). The above support the idea of promoting the use of specific agonists of estrogen receptor β such as diarylpropinitrile that inhibit the viability of colon tumor cells *in vitro*.

On the other hand, the substitution with E2 increased the number and size of tumors, as well as the TBS, when compared to Gx animals. This suggests a dimorphic mechanism of action for this hormone. Additionally, CRC patients have lower levels of T4, and increased aromatase levels result in higher intratumoral E2 concentrations. Overexpression of aromatase is associated with adenocarcinoma differentiation. More studies are necessary to fully elucidate the dimorphic role of E2 in males and females. It is important to mention that in this study, we evaluated a single dose for reconstitution and hormonal replacement. This leads us to think that the dimorphic effect of E2 could depend on the concentration and form of administration. In other studies with C57BL/6 male mice, this hormone had a protective effect when administered intraperitoneally at a very high dose (10 mg/kg every 7 days) (33). On the other hand, in a female PIRC rat model that develops tumors spontaneously, estradiol had no effect on tumor development when administered via slow-release pellets at a low dose (∼4 μg/kg/day) (31). This hormone could be modulating the tumor microenvironment differently as its effect on target cells depends on the dose. For instance, at high doses, E2 generally has an immunosuppressive effect, whereas low doses promote the production of proinflammatory cytokines (50). Interestingly, aromatase (the enzyme that converts T4 to E2) is overexpressed in the colon of men with colorectal cancer compared to healthy individuals (8). Furthermore, the expression of alpha estrogen receptors increases in tumor tissue compared to normal mucosa in men but not in women (7). The expression of this receptor is associated with a poor prognosis in CRC patients (51). This leads us to consider that another explanation for the dimorphic effect of estradiol, besides its concentration, is that it depends on receptor expression in the tumor. For example, in breast cancer, estrogen receptor beta is poorly expressed in breast tumors; however, it is associated with better prognosis and reactivation of the anti-tumor immune response (52). Activation of this receptor inhibits the cell cycle and also reduces the formation of angiogenic factors (53).

Regarding the mechanism, *in vivo*, E2 affects important pathways associated with inflammation and colonic tumor development such as NF kB, NRF2 and NLRP3. *In vitro*, the study with different tumor cell lines has shown the activation of pathways such as MAPK and Wnt/β-Catenin through the β estrogen receptor that inhibit the proliferation of tumor cells (33, 54).

On the other hand*, in vivo*, T4 increases the expression of some inflammatory factors such as MPO, IL-1, COX2 and INOS that are associated with the development of colorectal cancer. *In vitro*, this hormone modulates the Akt pathway, influencing cell adhesion and migration, either promoting or inhibiting it through the intracellular or extracellular androgen receptor, respectively (34, 55).

In our study, we observed no significant effect on the progression of colon cancer cell lines when treated with DHT. This suggests that the impact of these hormones might be mediated through their interaction with other cells in the tumor microenvironment, such as immune cells, stromal cells, nervous system cells, or microorganisms in the intestinal microbiota. On the contrary, our results indicate that E2 inhibits the proliferation of HCT116, a cancer cell line derived from males. Interestingly, this hormone promotes tumor growth in male animal models. It would be important to conduct a broader evaluation of the effect of estradiol and dihydrotestosterone on other cell lines derived from tumors of both men and women, as well as from non-neoplastic tissue. There are no studies in the literature on cell lines with this dimorphic focus. Additionally, this focus could help us begin to understand the discrepancies between the *in vitro* analyses conducted in this publication and the *in vivo* results that we obtained. On another hand, it would be worthwhile to investigate the effects of sex in other systems, such as the production of cytokines, chemokines, and neurotransmitters that are so important in the microenvironment of colon tumors.

Recently, our group conducted a review that specifically addresses the role of sex steroids in the pathophysiology of CRC, focusing on their effects on immune system cells, neurons, and their soluble factors (neurotransmitters and cytokines) (56). All these factors interact within the tumor microenvironment and play a significant role in the development of colorectal tumors (56). Our study revealed that E2 has an anti-inflammatory effect on the innate immune response, especially in studies that directly assess inflammation in colonic epithelium tumors. However, it is important to mention that estrogens can activate dendritic cells and lymphocytes, which are essential cells for tumor elimination, and their infiltration into the microenvironment is clearly associated with a favorable prognosis. E2 affects the enteric system. Primarily, it stimulates neurogenesis and maintains tissue architecture. Damage to enteric glial cells is a consequence of cancer invasion, and this damage leads to the release of prosurvival and proliferative signals that create a microenvironment favorable for tumor growth. Furthermore, although the effect of the microbiota is complex, E2 increases bacterial diversity, which is associated with a good prognosis in patients with colorectal cancer and reduces tumor progression in animal models by their interaction with ER-β. Apart from CRC, to our knowledge there are no studies that dimorphically study the effect of estradiol on other types of cancer (56).

Conversely, androgens play an immunosuppressive role in both the innate and adaptive immune systems. These findings suggested that these molecules help tumors evade immune responses. Additionally, although studies suggest that androgens do not alter neurogenesis, they can stimulate the synthesis of molecules such as those in the sympathetic nervous system (adrenaline and noradrenaline), 5-HT, Ach, and their receptors. These molecules promote tumor cell proliferation, tissue degradation, tumor formation, and the production of angiogenic factors such as MM-9 and 3. Furthermore, they stimulate the secretion of IL-12, IL-17, and IFN-γ, which potentially promote carcinogenesis through chronic inflammation. In addition, males have a lower diversity of microbiota than females, and animal models suggest that androgens favor a decrease in biodiversity, while the microbiota increases the bioavailability of androgens in the colon. Decreased diversity is a risk factor associated with poor prognosis in the early stages of CRC carcinogenesis (56).

In other types of cancer, we can observe the clear pro-tumoral role of androgens in the development of these diseases. Epidemiologically, men have a higher risk of almost all non-sex-dependent cancers. For example, high levels of T4 and DHT are associated with an increased risk of developing lung cancer (57). Moreover, androgen receptor knockout mice develop smaller tumors, and these hormones polarize lung macrophages toward an M2 phenotype that enhances tumor growth through immune suppression (58). Another example is seen in a mouse model of bladder cancer, where the percentage of males with tumors is higher than females, and DHT increases tumor growth and lung metastasis in males (not tested in females) (59). Androgens also play a significant role in breast cancer, traditionally associated with estrogens. In this cancer, they play a dual role by increasing proliferation in estrogen receptor-positive breast neoplastic cells and promoting it in triple-negative tumor cells (60). These examples underscore the importance of studies evaluating the role of androgens in tumor development in both sexes. Furthermore, it’s important to highlight their generally immunosuppressive effect, suggesting they prevent the immune system from eliminating tumors (61).

## 5 Conclusions

Epidemiological analysis show that sex is a statistic risk factor associated with CRC in men. Our results indicates that androgens have a protumoral role while estradiol have a dimorphic effect. In females this hormone protects and in males stimulate the development of tumors. The molecular and cellular mechanisms are not dilucidated in this work. However, our group is working to analyze the effect of sex steroids on the tumor microenvironment that involves cytokines, chemokines, neurotransmitters and microbiota.

## Data availability statement

The raw data supporting the conclusions of this article will be made available by the authors, without undue reservation.

## Ethics statement

All experimental procedures in the animals were approved by the Institutional Care and Animal Use Committee (Full name: Consejo para el Cuidado y Uso de Animales de Laboratorio, CICUAL), adhering to Mexican regulation (NOM-062-ZOO-1999), in accordance with the recommendations outlined in the National Institutes of Health (NIH) of the United States of America (Guide for the Care and Use of Laboratory Animals). The animal care and experimental practices were conducted at Unidad de Modelos Biológicos (UMB) in the Instituto de Investigaciones Biomédicas (IIB), Universidad Nacional Autónoma de México (UNAM). All experimental procedures in the animals were approved by the CICUAL, ID Number 6298. The present study adheres to the ARRIVE guidelines. For tumor induction, we utilized the gold standard protocol for inducing colorectal tumors (check material and methods). Throughout the study, the animals were monitored daily to promptly identify and alleviate signs of pain or distress.

## Author contributions

YR: Formal analysis, Investigation, Methodology, Visualization, Writing – original draft. LT-V: Conceptualization, Formal analysis, Supervision, Writing – original draft. KN: Data curation, Formal analysis, Funding acquisition, Investigation, Resources, Writing – original draft. VD: Investigation, Validation, Visualization, Writing – original draft. CG: Investigation, Methodology, Project administration, Visualization, Writing – original draft. JM: Conceptualization, Formal analysis, Funding acquisition, Supervision, Writing – original draft, Writing – review & editing.
